# Ultrasonic Guided Wave Testing on Cross-Ply Composite Laminate: An Empirical Study

**DOI:** 10.3390/s20185291

**Published:** 2020-09-16

**Authors:** Gerardo Aranguren, Josu Etxaniz, Sergio Cantero-Chinchilla, Jose M. Gil-Garcia, Muhammad Khalid Malik

**Affiliations:** 1 Faculty of Engineering in Bilbao, University of the Basque Country, 48013 Bilbao, Spain; josu.etxaniz@ehu.es; 2 Institute for Aerospace Technology & The Composites Group, The University of Nottingham, Nottingham NG7 2RD, UK; Sergio.CanteroChinchilla1@nottingham.ac.uk (S.C.-C.); Muhammad.Malik@nottingham.ac.uk (M.K.M.); 3 Faculty of Engineering Vitoria-Gasteiz, University of the Basque Country, 01006 Vitoria-Gasteiz, Spain; jm.gil-garcia@ehu.es

**Keywords:** structural health monitoring, ultrasonic guided wave testing, piezoelectric wafer active sensors, composite material

## Abstract

Structural health monitoring comprises a set of techniques to detect defects appearing in structures. One of the most viable techniques is based on the guided ultrasonic wave test (UGWT), which consists of emitting waves throughout the structure, acquiring the emitted waves with various sensors, and processing the waves to detect changes in the structure. The UGWT of layered composite structures is challenging due to the anisotropic wave propagation characteristics of such structures and to the high signal attenuation that the waves experience. Hence, very low amplitude signals that are hard to distinguish from noise are typically recovered. This paper analyzes the propagation of guided waves along a cross-ply composite laminate following an empirical methodology. The research compares several implementations for UGWT with piezoelectric wafer active sensors. The reference for comparison is set on a basic mode, which considers the application of nominal voltage to a single sensor. The attenuation and spreading of the waves in several directions are compared when more energy is applied to the monitored structure. In addition, delayed multiple emission is also considered in multisensor tests. The goal of all the UGWT configurations is to transmit more energy to the structure such that the echoes of the emission are of greater amplitude and they ease the signal processing. The study is focused on the realization of viable monitoring systems for aeronautical composite made structures.

## 1. Introduction

The use of carbon fiber reinforced polymer (CFRP) composite structures is becoming widespread in the aerospace field as they are generally stronger and lighter than metals [1]. Simultaneously, the need for safety and reduced maintenance costs is driving the development of structural health monitoring (SHM) systems that monitor the condition of any structure throughout its life. The development of SHM techniques started more than two decades ago [2,3]. Nowadays, they are applied to metallic or concrete bridges or similar structures [4]. However, its implementation faces great difficulties when applied to light structures in complex structural assets such as aircrafts [5], especially when they are made of CFRP. Ultrasonic guided wave tests (UGWT) are one of the most viable technologies for SHM, due to its active operation and the capability of the guided waves to interact with the entire section of the structure and cover large areas [6]. UGWT technology uses piezoelectric wafer active sensors (PWAS), embedded or permanently attached to the structure [7], to emit acoustic guided waves or Lamb waves throughout the structure. The acquisition and processing of these waves serves to detect defects that may arise in the structure throughout its life such as fracture, fatigue, delamination in the CFRP plate, or corrosion in metals [8,9]. Furthermore, UGWT technology is low cost, has low impact on structures, and the systems are low weighted. As a result, this technique shows a promising future in the aeronautical industry, where many thin-walled structures made of CFRP are used [10] and the optimal maintenance of structures is fundamental. 

The attenuation of guided waves in metals is low [11] and such waves can be used to monitor large parts or structures. The internal structure of metals is homogeneous and the sound propagates with the same physical properties along all axes, i.e., metals are isotropic materials. On the other hand, the structures made of CFRP are synthetically manufactured with heterogeneously arranged materials and/or threads. This feature makes the transmission of sound along the composite structure nonlinear, i.e., composite is an anisotropic material [12,13,14]. The nonlinear wave propagation plus the wide range of composite materials, the specific geometry of each structure, the number of wave propagation modes in multimodal ultrasonic guided waves [15], and the high attenuation [16] lead to performance close to Chaos Theory [17,18]. Lingfang et al. [19] have recently published a theoretical method to calculate the propagation of a guided wave on a composite material employing a unique PWAS. There are numerous papers that introduce research on the behavior of acoustic waves and propose the development of UGWT in structures made of CFRP material employing a unique emitting PWAS. For instance, Putkis et al. [20] describe the energy velocity, dispersion, mode coupling, and energy focusing factor of the guided wave propagation in composite material. Zagrai et al. [21] propose the use of UGWT with PWAS for space applications and consider an SHM system design based on a network of PWAS. Sharif-Khodaei et al. [22] propose the use of the Delay and Sum algorithm, which consists of adding the signals received in a phased-array of PWAS to increase the amplitude of the acquired signals and obtain information on the direction of the defect (it is also known as beamforming reception). Other authors used mixed techniques by combining UGWT with other equipment to compensate for the high attenuation of the composite material for the propagation of acoustic waves. For example, Leckey et al. [23] use a laser Doppler vibrometer. Murat et al. [24] apply laser interferometers for the measurement of guided waves. Pohl et al. [25] exploit the measure of the impedance spectroscopy. The techniques mentioned are useful for the inspection of parts in the laboratory or during manufacturing, i.e., for non-destructive tests (NDT). However, they are not viable for a real-world SHM system due to the complexity of the instrumentation used and the need for autonomous and real-time operation. To sum up, the literature relates the interest of academia in the tests with guided waves generated and acquired by PWAS to monitor structures made of CFRP material. Additionally, it also shows that the reported amplitude of acoustic waves is not enough to monitor the structures in real-world engineering scenarios due to the high attenuation of acoustic signals in CFRP material, which make them difficult to be distinguished in noisy environments. 

The goal of the research introduced here is to propose a new technique that enables the realization of UGWT for SHM in structures manufactured using composite material. This way, in the recent future, light-weighted electronic systems can be installed in aircrafts so that defects or accidents in the structures will be detected without expensive revisions carried out by human expert workers. The research compares the techniques to provide more acoustic energy to UGWT and, after the propagation of the waves along the plate, it verifies that valid signals with high enough amplitude to be processed are received. At the same time, this paper investigates the features of the propagation and spreading of guided waves in composite structures. 

The technique uses only PWAS as sensors and actuators so that it is feasible to be applied to aeronautical thin-walled structures made of CFRP material. Since the structures under test are nonlinear, an empirical methodology based on the performance of UGWT and the analysis of the results has been chosen [26]. Structures made of CFRP material can be built with several plies, orientations, and processes, making it difficult to generalize any rule. Even a certain specimen includes numerous irregularities at microscopic level [27] that gives it unique features. In this research, a cross-ply composite laminate is used to validate the techniques under study, with sheets at 0 and 90 degrees. The features of the commercial equipment used by many researchers are not enough to perform many of the tests proposed in this research. As a result, the types of UGWT considered here have been carried out with electronic equipment of specific design. 

In the investigation, the basic procedure has been compared with two new procedures: provide more energy through a single PWAS (procedure P1) and provide more energy using several PWAS (procedure P2).

The basic procedure is the simplest UGWT. It applies a nominal excitation signal of the greatest amplitude supplied by the electronic equipment to a single PWAS. The P1 procedure applies a signal of greater amplitude than the nominal one to a single PWAS. Since the impedance of a PWAS for a given frequency is constant, there will be an increase in the intensity and amplitude of the generated acoustic wave. The way to increase the voltage amplitude of the signal depends on the electronic circuitry used to generate the signal, such as an amplifier, or the series arrangement of signal generators.

The P2 procedure consists of the simultaneous transmission of signals through several PWAS, so that the interference among signals generates a combined signal with higher energy. It can be carried out by transmitting signals simultaneously through several PWAS or using the radar technique applied to guided waves, as described in [28]. This technique can be achieved by varying the amplitude or the starting moment of the signal emitted by each PWAS. These variations alter the interferences produced and, therefore, the waveforms propagated along the structure. In the study, the same signal is transmitted through all the PWAS, but a delay is added to the signal for each PWAS. Cantero-Chinchilla et al. [29] show a practical study of the application of this technique in aluminum specimens. They report the formation of a main beam with the technique. Therefore, the technique is named transmission beamforming. 

Section 2 presents the setup used in UGWT and the results of a basic UGWT that is considered as a reference for the other procedures. Section 3 presents and compares the two procedures to transmit more energy to the PWAS. Section 4 compares the propagation of acoustic waves in the two procedures and introduces the focused mode. Section 5 extends the former comparison to the spreading of the signals in different directions. The results of the tests are discussed in Section 6 and the most appropriate techniques are proposed to build SHM systems that use guided waves and are to be applied to composite structures. Finally, Section 7 summarizes the conclusions.

## 2. Ultrasonic Guided Wave Testing

### 2.1. Setup

**Figure 1:**
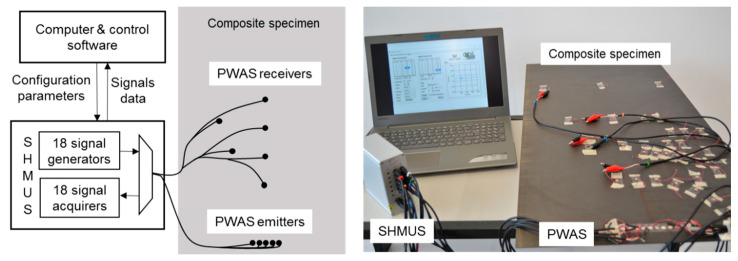
Schematic and photograph of the setup of the UGWT.

The setup for all the tests is shown in Figure 1. It includes several PWAS, a structural health monitoring ultrasonic system (SHMUS) to carry out the tests, and a computer to process the acquired signals. The SHMUS is the electronic equipment that generates the electrical excitation signals that are applied to the PWAS so that they transform them into acoustic waves. Moreover, it acquires the electrical signals that come from the waves received in the PWAS. Between generation and acquisition, acoustic waves propagate along the structure and are reflected at edges and obstacles and interact with defects. To determine the health of the structure and find defects that may arise over time, the electrical signals acquired are properly processed. 

The specimen used in the tests is a 800 × 400 mm 100% carbon fiber reinforced polymer (CFRP) plate and cross-ply of [0_2_/90_4_]s (12 plies of 0.15 mm, total 1.8 mm). Several PWAS of 7 mm in diameter and 0.2 mm thick (Steminc model SMD07T02R412WL) were glued onto the plate with epoxy. The position of the PWAS used in each type of test is described later. 

The SHMUS equipment is specifically designed for UGWT [30]. For this reason, its features are more oriented to UGWT than the ones of generic commercial equipment. For example, SHMUS can generate and acquire up to 18 signals simultaneously. Following the guidelines regarding the calculation of the signal length for UGWT described in [31], 3.5-cycle Hanning-windowed excitation signals were programmed for the UGWT in this paper. 

**Figure 2:**
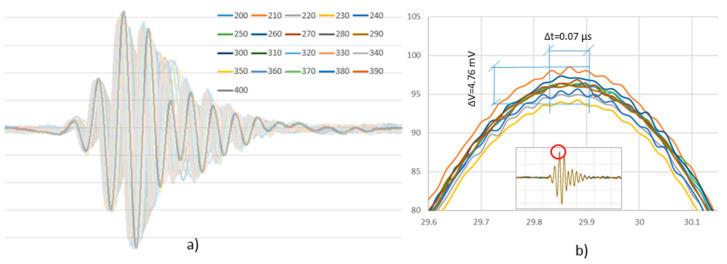
Results of the tests to determine: (**a**) the optimal frequency used in the UGWT, where the signal with the highest amplitude corresponds to the test at 340 kHz and it is highlighted; (**b**) the precision of the repeatability of the UGWT, and the focus is set on the peak of a cycle.

The optimal center frequency of 300 kHz was determined by considering the resonant frequency of the PWAS given by the manufacturer. Additionally, multiple UGWT at adjacent frequencies provide the fine tuning of the optimal center frequency. Two PWAS in the center of the plate 120 mm apart were considered in the tests. The frequency of the transmission signal ranged from 200 to 400 kHz in 10 kHz increments. The results of the tests are summarized in Figure 2a. The acquired signal with the greatest peak-to-peak amplitude is highlighted. It corresponds to the excitation signal of 340 kHz frequency. The optimum frequency is defined by the type of PWAS used, the material of the structure, and the gluing of the PWAS to the plate. This frequency value has been considered in all other tests.

To check the precision of the repeatability of the UGWT, 10 consecutive UGWT were carried out under the same conditions. Figure 2b shows the 10 complete signals measured and the detail of a maximum. As a result, it is found that the repeatability in the tests is as precise as 4.76 mV in amplitude and about 0.07 µs in time. The SHMUS acquires the data with a resolution of 12 bits (the least significant bit is equivalent to 0.238 mV) and with a sampling rate of 60 Msps (i.e., with a 0.0167 µs sampling period). These features and precision are kept in all the UGWT presented in the paper. 

### 2.2. Basic UGWT

**Figure 3:**
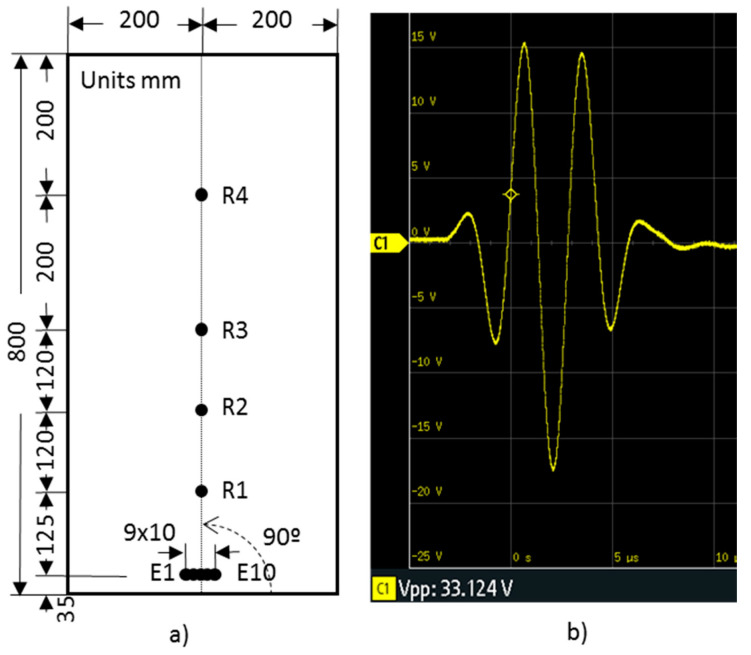
(**a**) Position of the PWAS for the basic UGWT and (**b**) excitation signal applied to the PWAS E5 for the basic UGWT.

The position of the PWAS for the basic UGWT is shown in Figure 3a. The PWAS E5 and R1–R4 were arranged in-line with the 8-ply at 90 degrees of the cross-ply plate. This is the preferred direction of propagation of acoustic waves, i.e., it is the direction that shows the smallest attenuation. The pitch-catch configuration was chosen in the UGWT. Concretely, here, the PWAS E5 is used to transmit the wave to the specimen and the propagated wave is acquired by the PWAS R1–R4. The peak voltage of the signal applied to E5 is 33.12 V. It is considered as reference or nominal for all other tests. Figure 3b shows the waveform applied to PWAS E5. 

**Figure 4:**
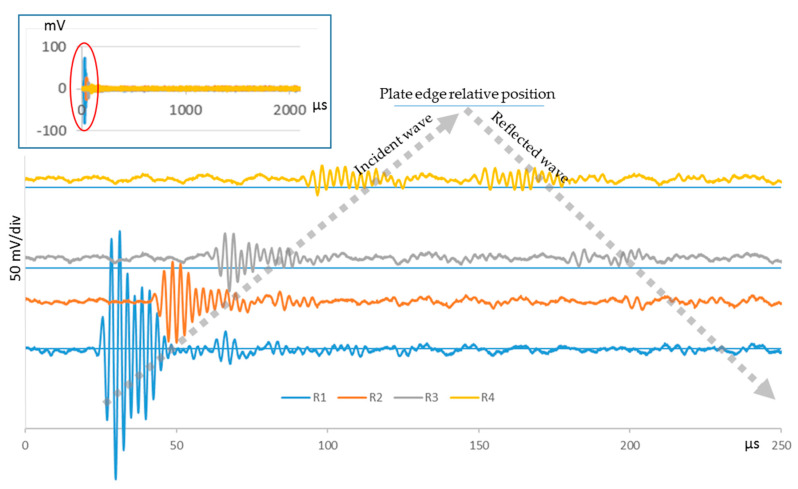
Signals acquired at R1–R4 during the basic UGWT. The upper part corresponds to the signal acquisition for 2 ms. The lower part corresponds to the significant part of the signals, when their amplitude is greatest. An offset proportional to the distance between adjacent PWAS is added to each signal to help understanding.

Figure 4 shows the signals acquired in the PWAS during the basic UGWT. Direct propagation, from the emitting PWAS towards the opposite end of the structure through R1 to R4, and the reverse propagation from R4 to R1, after the reflection at the edge, are distinguished. 

The data shown in Table 1 were measured and calculated to analyze the propagation of waves along the plate. The second column sums up the distance that the acoustic wave front traveled from the PWAS E5 to each PWAS (from R1 to R4), as well as the distance that the wave front reflected on the opposite edge of the plate traveled from the edge to each PWAS (from R4 to R1). The third column summarizes the maximum amplitude of the received wave, measured between two consecutive and opposite maximum peaks. The fourth column shows the time-of-flight (ToF), i.e., the time it takes for the acoustic wave to reach each PWAS, measured in the zero-crossing between the first two cycles. The fifth column provides the results for the relative velocity, which is calculated as the ratio between the distance traveled and the ToF between two consecutive PWAS. The absolute speed, shown in the sixth column, is calculated as the ratio of the total distance and ToF. The frequency summed up in the seventh column is calculated as the inverse of the time between two consecutive peaks in the signal acquired in the PWAS. 

**Figure 5:**
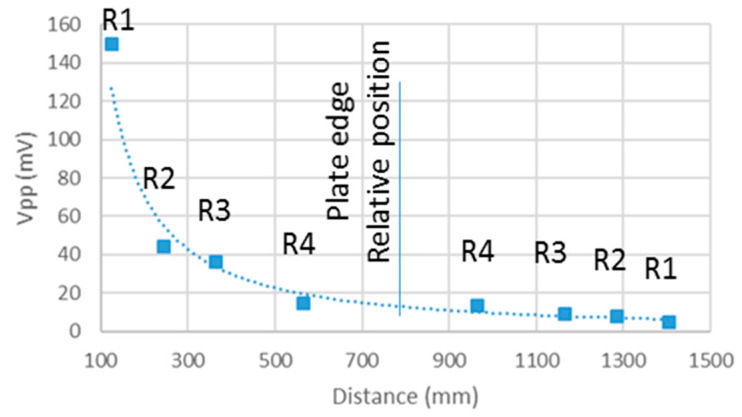
Peak-to-peak voltage measurements of the waves acquired in each PWAS on the direct path along the specimen (R1 to R4) and on the path after the reflection on the opposite plate end (R4 to R1).

The data summarized in Table 1 are the basis for Figure 5, which shows the maximum peak-to-peak amplitude of the signals measured at each one of the PWAS on the direct and reflected paths. 

The data provided by the test show that the attenuation of the acoustic wave is strong due to the type of material and it decreases when distance increases. After travelling across the plate and being reflected at the opposite end, the amplitude of the acoustic wave acquired on the return path is very low and can be easily confused with noise. This fact makes it difficult to extract information to monitor the health of the structure.

The test results show that the group propagation speed remains fairly stable for the direct path, about 6900 m/s. The frequency of the signal acquired in the PWAS is also kept stable at the center frequency of 340 kHz. However, the low amplitude of the wave during the reflected path prevents group speed or frequency from being measured. 

The basic test was carried out with a 33 peak-to-peak volts excitation windowed Hanning signal, higher than the ones used in most of the UGWT presented in the references. Despite using such amplitude and a small specimen, the amplitude of the signal along the reflected path is not high enough to be processed and monitor the health of the structure. Thus, the UGWT can be used to monitor structures, but the amplitude of the acoustic signals used must be increased.

## 3. Enhanced UGWT Procedures

The most obvious method to improve the results of UGWTs in composite specimens is to increase the energy transmitted in the tests. This increases the amplitude of the reflected waves, which eases the subsequent analysis to determine the health of the structures. 

In this section, two procedures are introduced and tested. They provide three times the nominal power used in the basic UGWT. The procedures are compared over the same specimen and with the PWAS layout considered in the basic UGWT (Figure 3a). 

**Figure 6:**
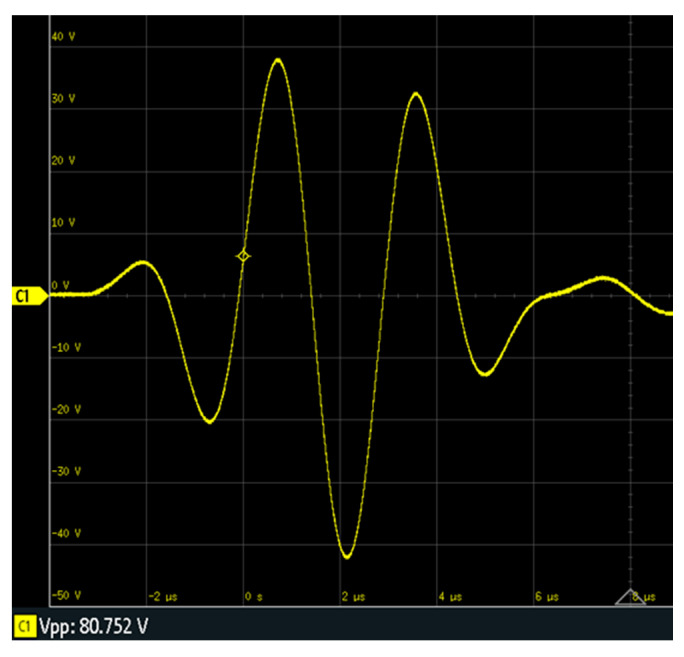
Signal applied to E5 in the UGWT according to procedure P1.

The first procedure (P1) consists of transmitting more acoustic energy to the specimen by applying higher voltage to a single PWAS. Figure 6 shows the waveform (80.75 Vpp) measured in E5 when it is applied to the emitter PWAS E5. It is equivalent to three times the nominal power used in the basic UGWT. 

The second procedure (P2) consists of applying the original signal of the basic UGWT (Figure 3b) to each one of the three PWAS considered, namely, PWAS E4, E5, and E6 of the layout shown in Figure 3a, which are 10 mm apart. 

**Figure 7:**
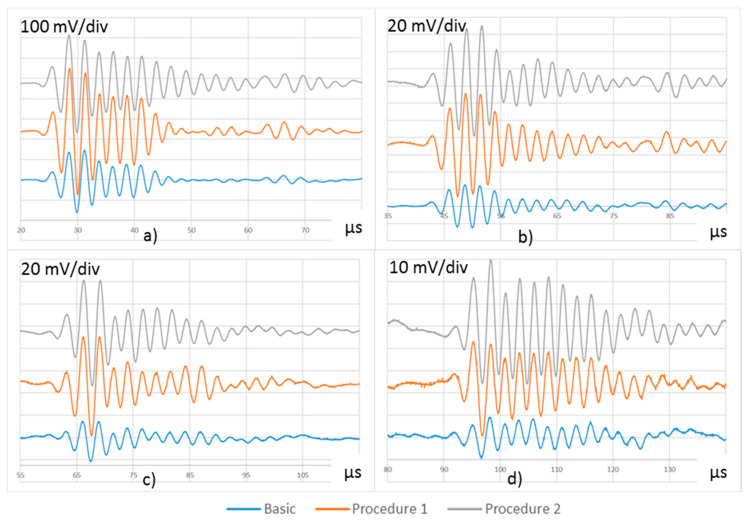
Waveforms acquired in (**a**) R1, (**b**) R2, (**c**) R3, and (**d**) R4 with the following UGWT procedures: basic, P1, and P2. The figures include vertical offsets for easy viewing.

UGWT were carried out with the basic, P1, and P2 procedures, on the described setup. Figure 7 shows the waveforms acquired on PWAS R1 to R4. 

Table 1 and P2 procedures provide the tests with three times the nominal energy. The amplitude of the waveforms received at the four receiving PWAS is approximately three times the one measured during the basic test. The P1 and P2 procedures produce very similar waveforms along the four PWAS, and they can be considered equivalent procedures to monitor the direction defined by PWAS R1 to R4.

**Figure 8:**
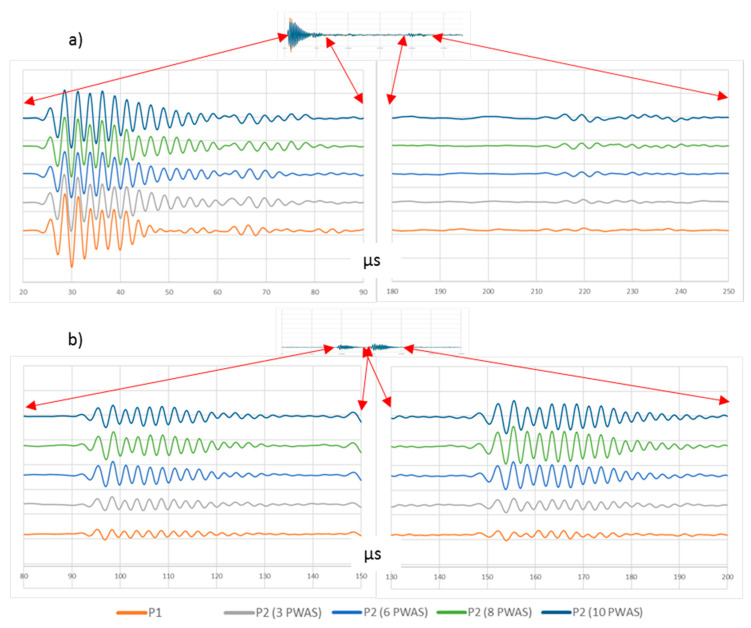
Signals obtained in PWAS (**a**) R1 and (**b**) R4 in UGWT according to procedures P1 and P2 using 3, 6, 8, and 10 PWAS as wave emitters. In all cases, the vertical scale is 100 mV/div.

The voltage that can be applied to any PWAS is limited by the maximum voltage that piezoelectric transducers can withstand as well as by the maximum voltage that the electronic circuits included in the setup can generate. As a result, there is a limit for the P1 procedure. The piezoelectric transducer manufacturer gives the maximum voltage that can be applied to the PWAS without breaking it, about 150 volts. On the contrary, more than one PWAS is used in the P2 procedure. Then, more acoustic energy can be applied to the specimen without jeopardizing the integrity of the PWAS. As a result, greater amplitude waves that provide greater range can be obtained. To demonstrate it, new UGWTs were performed with the same setup and more PWAS: 6 (E3–E8), 8 (E2–E9), and 10 (E1–E10). Figure 8 shows the waveforms measured in PWAS R1 and R4. 

**Figure 9:**
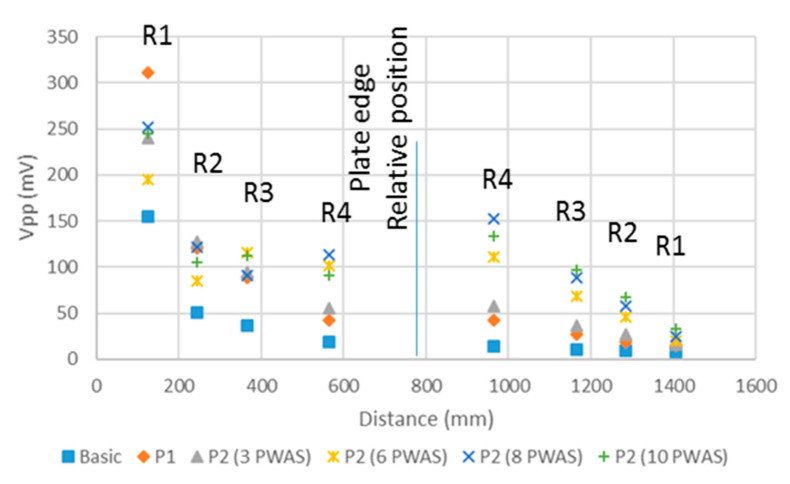
Peak-to-peak voltage of the waves acquired in each PWAS along the direct propagation path and the path of the wave reflected in the plate edge (according to the different UGWT procedures tested).

Table 2 and Figure 9 ease the comparison among the amplitudes of the waves measured in the procedures considered, i.e., the basic one, and procedures P1 and P2 with different sets of PWAS.

As the test results state, the more power applied to the emitting PWAS, the higher the wave amplitude measured in the set of receiver PWAS is. This phenomenon eases the processing of the measured wave because the noise can be discarded easily. The effect of using multiple PWAS as emitters (procedure P2) is similar to the application of higher voltage in a single PWAS (procedure P1). However, in procedure P2, the waves from each PWAS combine their energy along the different propagation paths. Along the direct propagation, the wave amplitudes measured in R1 and R2 are almost similar when using procedures P1 and P2 with 6, 8, and 10 PWAS. However, when the distance of direct wave propagation increases over a certain point, i.e., in R3 and R4, the use of a greater number of PWAS produces an increase in the acquired wave amplitude. After the direct wave is reflected in the plate edge, the set of PWAS acquires signals of greater amplitude in the tests that use multiple emitting PWAS. This is because they keep on adding the emitted signals. At the end of the propagation, signals of the amplitudes proportional to the emitted energy are acquired in R3, R2, and R1, because the distance traveled makes the distance among the different emission points negligible, and all the energy emitted for the test is combined. 

## 4. Wave Propagation

In composite structures, the fiber directions favor the propagation of the guided waves. In order to complete this study, additional tests were carried out not following the directions of the fibers. A first UGWT set was designed to study the wave propagation with the distance. A second set of tests is focused on the wave spreading in different directions (see next section).

**Figure 10:**
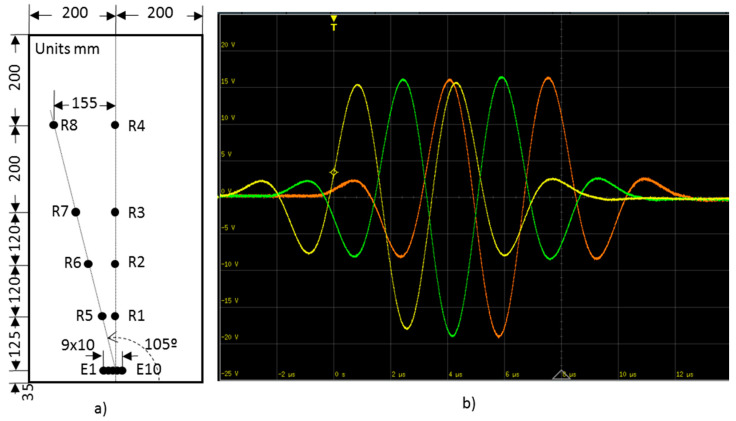
(**a**) Arrangement of emitters and receivers on the specimen. (**b**) Signals applied to E6 (yellow), E5 (green), and E4 (orange) according to the P2 procedure with 0.16 µs delay.

In order to analyze the propagation with the distance, the direction of 105° was chosen. The direction is sufficiently separated from the fiber direction and allows the setup of four PWAS inside the specimen, as shown in Figure 10a. UGWT techniques based on procedures P1 and P2 are compared, but now, out-of-phase signals with programmable delay are considered to assess the delayed multiple emission technique. Specifically, a 0.16 µs delay step between the adjacent emitted signals was added. The value was set after the following test. Figure 10b shows an oscilloscope capture of the signals synthesized for the three PWAS considered in a P2 procedure-based test.

**Figure 11:**
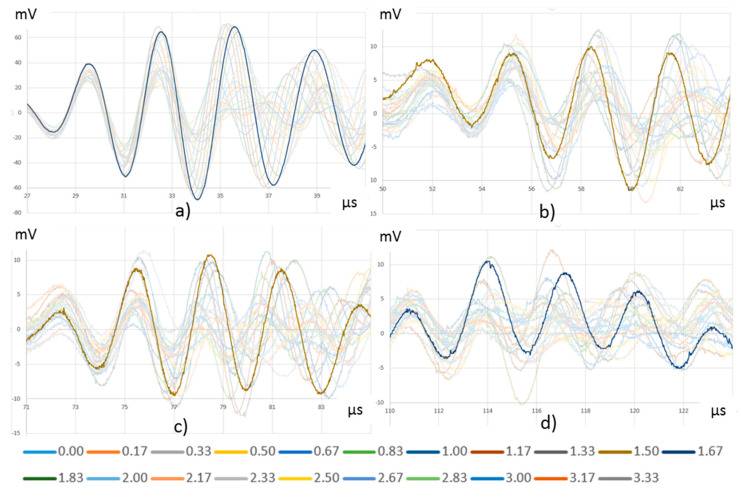
Acquired signals in PWAS (**a**) R5, (**b**) R6, (**c**) R7, and (**d**) R8 as function of the excitation signal delay applied to E6–E4. The received highest peak-to-peak amplitude for each PWAS is highlighted.

The theoretical calculation of the delay between adjacent driving signals to focus the transmitted energy on a particular direction has many difficulties due to the nonlinear behavior of composite materials and the use of a particular specimen. Then, the delay was determined empirically. With the described setup, 21 UGWT were performed following procedure P2 with three emitting PWAS (E4, E5, and E6) and delaying the signals applied from 0 to 2 µs in 0.167 µs steps. The R5 to R8 PWAS were chosen to acquire the received waves. Figure 11 represents the significant part of the recorded signals in each PWAS for the 21 tests. They show that the delay of the emitted signals produces a variation in the amplitude and phase of the acquired signals due to the different interference construction. The signal with the largest peak-to-peak amplitude has been highlighted. It corresponds with 1.50 and 1.67 µs delays. A more precise test, not shown in this paper, determined that 1.60 µs delay provide the highest amplitudes in reception. This set of tests shows that the delayed multiple emission technique on composite material does not create a main narrow beam, as it does on isotropic materials, while it is named the transmission beamforming technique [29]. All in all, there is a delay that originates the maximum amplitude in a given direction.

**Figure 12:**
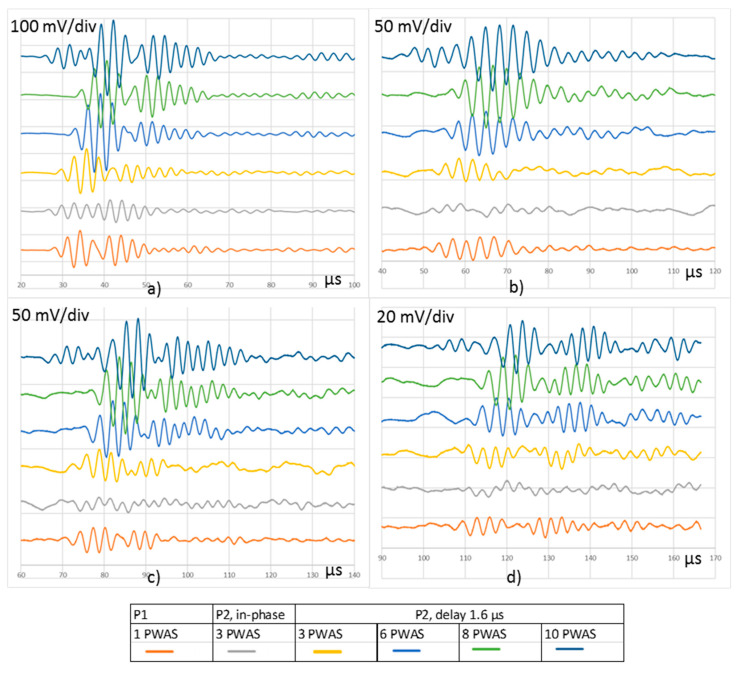
Signals acquired in (**a**) R5, (**b**) R6, (**c**) R7, and (**d**) R8 following different procedures. An offset was added to each signal for easy display.

New UGWT tests to compare the different procedures were carried out. The basic procedure and P1 were repeated under the same conditions as the previous tests because they can only be run in one way. The procedure P2 was ran in two ways. Firstly, three in-phase emitter PWAS were considered, and secondly, three, six, eight, and ten emitter PWAS with a delay of 1.60 µs to focus the acoustic energy on the 105° direction. The comparison is based on the signals acquired in R5 to R8 receiver PWAS (Figure 12).

It can be deduced from the tests represented in Figure 12 that the use of several in-phase PWAS, focused at 90°, produces low amplitude signals in the reception for the 105° direction, due to the destructive interference among the emitted waveforms. Conversely, several emitter PWAS focused on a given direction by means of the application of proper delay produce signals with higher amplitudes as their interferences are constructive. Except for the test focused to 90°, i.e., with in-phase signals, the amplitude of the received signals keeps certain proportionality with the emitted energy. The signals obtained in the procedure P1, and those obtained in procedure P2 with three PWAS focused on 105° produce similar signals in reception. As shown, when more emitter PWAS that focus on the same direction following procedure P2 are used, the amplitude of the acquired signals along the 105° direction increases. 

## 5. Wave Spreading in Different Directions

**Figure 13:**
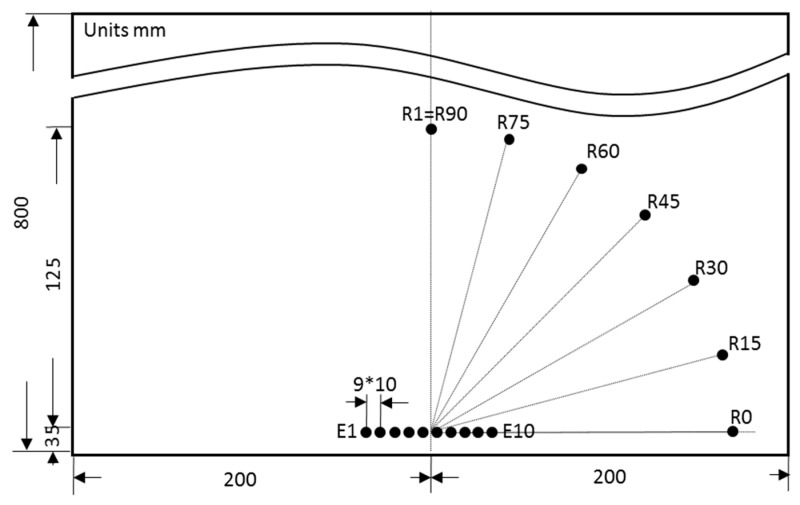
Arrangement of PWAS on the specimen to study wave spreading on different directions.

To study the wave spreading in some directions, several PWAS were arranged in an arc around the middle of the emitting phased array. The directions considered were 75°, 60°, 45°, 30°, 15°, and 0° (the PWAS were named R75 to R0 respectively in Figure 13). Additionally, R1 PWAS was renamed R90 for this study. UGWT tests were carried out following procedures P1 and P2 with ten emitting PWAS and several emitting delays.

**Figure 14:**
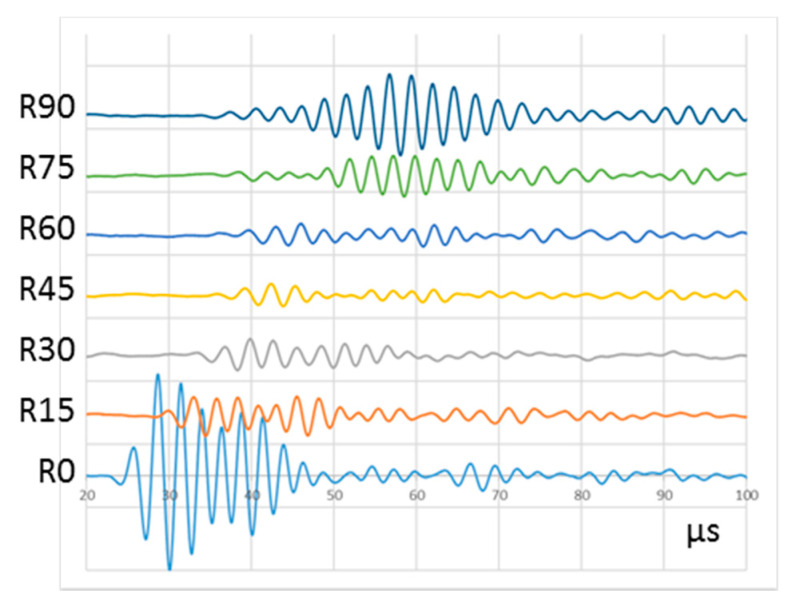
Signals acquired in R0 to R90 following procedure P1. An offset was added to each signal for easy display. Vertical scale is 100 mV/div.

**Figure 15:**
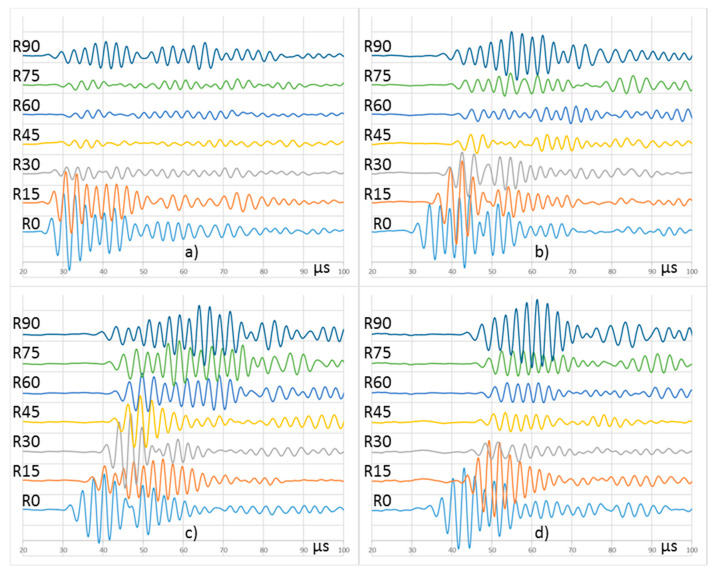
Signals acquired in R0 to R90 following procedure P2 when different delays are applied to the emitting PWAS E1 to E10: (**a**) 0.33 µs, (**b**) 1.60 µs, (**c**) 1.80 µs, and (**d**) 2.33 µs. An offset was added to each signal for easy display. Vertical scale is 100 mV/div.

Figure 14 shows the signals acquired by all PWAS in the arc when following procedure P1. Since only one emitting PWAS is driven, the test can only be run under the same conditions and therefore, it provides a unique result. Conversely, UGWT tests performed following the procedure P2 can be run applying several delays to the signals emitted by the PWAS. Figure 15 shows the results of four UGWT with different delays in the emitted signals. Table 3 compares peak-to-peak voltages measured with procedure P1, and procedure P2 with ten PWAS and a set of delays. 

The UGWT carried out following procedure P1 shows the typical anisotropic attenuation [32] of a cross-ply composite laminate structure. Here, the fiber layout favors acoustic wave propagation in 0° and 90° directions, whereas the signal suffers higher attenuation in the intermediate directions. On the other hand, the UGWT tests performed following procedure P2 and different delays provide different results. The UGWT run with in-phase signals provide similar results to those obtained in the procedure P1, i.e., the highest amplitudes happen in two directions, 0° and 90°. The tests run with wave emissions delayed from 1.6 to 2.0 µs produce higher amplitudes in the intermediate directions, which corresponds to the energy focusing on emission. All signals acquired in the 0° direction obtained in all the tests performed after procedure P2 show remarkable amplitude, especially when tests are focused on that particular direction.

The ToF of the wave front also varies with the delay and PWAS. In a cross-ply composite laminate, the propagation speed of the first symmetric mode, S0, is predominantly faster in the direction of the fibers. This can be confirmed in Figure 15, where signals reach R0 and R90 faster than the rest of the PWAS located along the arc. The increase in the delay of the emitted signals in all the tests means a bigger ToF of the wave front.

## 6. Discussion of Results

UGWT on composite specimens is not easy to carry out because the acoustic waves show nonlinear performance due to the heterogeneity of the material. In addition, the results obtained in UGWT on composite specimens cannot be extrapolated due to the specific design of their plies. Therefore, the results summarized previously can be applied only to tests under the same conditions and type of specimen. However, some results can be useful for other setups and for the development of UGWT systems for composite structures.

The attenuation for acoustic waves propagating along composite specimens is relevant. Furthermore, it depends on the direction of propagation of waves. The least attenuation happens in the direction(s) of the fiber(s) inside the internal structure of the specimen. However, the results of the UGWT summarized here show that the more energy is transmitted, the higher amplitude of the wave propagated.

The procedure considered to transmit more energy with a single PWAS, i.e., procedure P1, achieves the increase in the amplitude of the acquired wave according to the increase in the energy transmitted. P1 is limited to the maximum voltage that can be applied to a single PWAS. Additionally, there is only one way to perform P1 UGWT, i.e., there are no more parameters to tune to maximize the amplitude of the wave acquired. The results of UGWT following procedure P1 show good results for wave propagation in the directions of the fibers, but the attenuation is much larger in other directions (Figure 14). Hence, the monitoring of a specimen with procedure P1 provides information about its health in the directions of the fibers, but if flaws happen in other directions, the signals acquired will not be notoriously modified and flaw detection will be hard to achieve.

Procedure P2, which employs several PWAS, allows for more energy to be transmitted without jeopardizing their integrity. Their use with delayed multiple emission techniques does not create a main beam, as it does in any material with isotropic behavior [29], but it allows for multiple tests in different directions where the acquired signals show different waveforms (Figure 15). The wave front can be focused on specific direction by tuning the delay between adjacent PWAS. For example, Table 3 shows that a maximum voltage R75 is achieved with an emitting delay of 1.6 µs, whereas the wave front is focused on R60 when the delay is 1.8 µs. The UGWTs depicted in Figure 12 to measure focus at 105 and 75 (R75) degrees have been performed using the same 1.6 µs delay, since there is symmetry about the 90 degrees axis. However, the emissions have been synthesized to target quadrant 1 or quadrant 2 by reversing the order of the emitting PWAS. If an emitting PWAS array is lined up at the center of a symmetric plate, it will not be able to be distinguished between quadrants 1 and 2 and quadrants 3 and 4.

The acquired waveforms contain information about the material, the specimen, and its structural health. These waveforms do not change as long as the UGWT test is performed under the same conditions and the structure suffers no modification. Damages suffered by the structure throughout its life modify these waveforms. UGWT tests performed following the procedure P1 provide only one result, i.e., one waveform that has to be analyzed to monitor the structure. The signals acquired following the procedure P2 depend on the focus considered as its information comes from different paths of the waveform. Therefore, they can monitor different areas of the structure. The results of the tests shown in Figure 9 and Table 2 illustrate that the delayed multiple emission produces wave propagation following different paths. Once the waves are reflected at the plate edges, they continue with the interferences and the amplitude of the acquired signals may be increased. The different propagation paths can be considered as a wave front that can monitor a stripe of the structure in the focusing direction.

**Figure 16:**
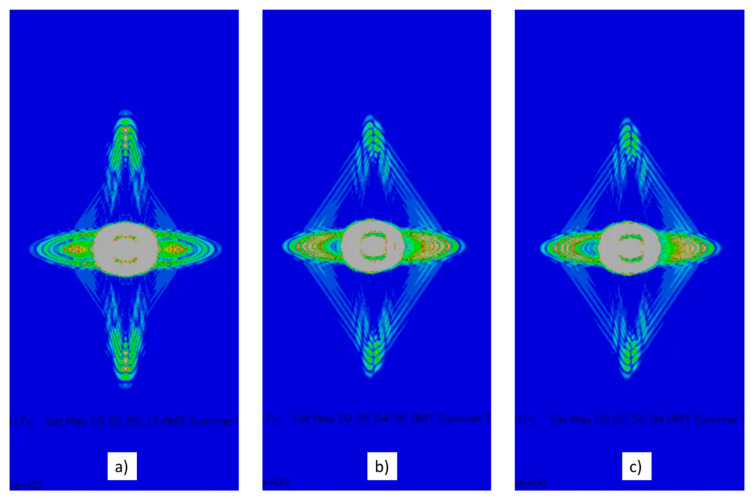
Images captured at 20 µs after the beginning of the simulation of the waveforms propagated on a composite material when they are generated by two PWAS and the emission is delayed (**a**) 0 µs, (**b**) 1.6 µs, and (**c**) 2.0 µs.

In order to confirm the empirical results, the propagation of acoustic waves generated by two emitter PWAS on a cross-ply composite laminate with the same layout as the empirical study was simulated. The PWAS were located at the plate center to avoid edge reflections. As far as possible, similar parameters as those found or measured in the tests were used. Figure 16 shows an instant of three simulations: the simulation without delay, with 1.6 µs delay, and 2.0 µs delay.

All images in Figure 16 show how the propagation is boosted by the layout of the fibers in 0° and 90°. The greatest wave amplitude happens in the 0° direction because the wave fronts line up with the emitting PWAS. Figure 16a displays symmetry of the wave fronts around the 0° and 90° directions, i.e., the horizontal and vertical axes. The interference among the emitted waves produces such maximums in amplitude in both directions. The wave reflected in the plate edge travels back to the array of PWAS. On the contrary, Figure 16b,c do not display a unique wave front but two. The wave fronts follow symmetric paths separated a certain angle from the 90° direction. The delay applied to the array of PWAS defines the direction of the path followed by the wave front. The sign of the delay determines which one of the two wave fronts is bigger and travels faster. The wave fronts reflected in the plate edge do not travel back to the array of PWAS, but they travel around the whole plate. 

Delayed multiple emission increases the relative amplitude of the wave front in the non-primary directions when some delay is added. The experimental results corroborate it (Figure 15). Thus, the inspection of areas off the primary directions can only be performed following procedure P2, whereas procedure P1 or the basic one could not explore those areas, leading to a biased or incomplete SHM system.

## 7. Conclusions

Different procedures to deliver more energy in the UGWT on composite material to monitor its structural health have been analyzed. These procedures consist of applying higher voltage to one single PWAS or using a delayed multiple emission technique on several PWAS. The second procedure is a novel technique and requires the development and use of a device with the capability to carry out this kind of test.

It has been shown that supplying more energy to an UGWT creates signals with higher amplitude. This fact provides measurements with greater signal-to-noise ratio, which supports its usability in SHM systems. The use of several PWAS to monitor composite structures has been validated. This technique produces wave amplitudes similar to those found in UGWT tests with one PWAS that use the same amount of energy. In addition, this technique provides the advantage of saving the integrity of the PWAS.

Moreover, the use of multiple PWAS along with the delayed multiple emission technique focuses the emission of waves and helps the alignment of the wave front with certain directions. This technique provides the acquisition of higher amplitude waves, even in the non-primary directions. Consequently, each emitted wave and its reflections in the edges of the plate can travel further and, thus, eventually, the whole specimen can be monitored.

An empirical methodology has been applied in this research and it has been validated on a given cross-ply composite laminate, but it can be presumed that the conclusions drawn here can be extrapolated to other types of composite materials.

## Figures and Tables

**Table 1:** Magnitudes measured in the PWAS and calculated during the basic UGWT.

	PWAS	Distance (mm)	Amplitude pp (mV)	ToF (µs)	Relative Speed (m/s)	Absolute Speed (m/s)	Frecuency (kHz)
	E5	0	33124.00	12.43		6901.83	340.00
Direct propagation	R1	125	149.71	30.57	6891.68		357.51
	R2	245	44.03	48.03	6872.98		352.87
	R3	365	36.18	65.32	6939.19		345.68
	R4	565	14.75	94.30	6903.28		328.19
Reflected propagation	R4	965	13.33	The signals amplitude is not enough for measurement or calculation			
	R3	1165	9.05				
	R2	1285	7.62				
	R1	1405	4.99				

**Table 2:** Peak-to-peak voltage of the signals acquired in R1 to R4 PWAS along the direct and reflected propagation paths with the different test procedures.

Vpp (mV)		Process					
		Basic	P1	P2 (3 PWAS)	P2 (6 PWAS)	P2 (8 PWAS)	P2 (10 PWAS)
Direct propagation	R1	154.46	311.30	240.62	194.92	252.28	245.38
	R2	50.46	120.19	128.04	84.73	122.09	104.96
	R3	36.18	89.01	94.96	116.38	90.68	111.86
	R4	18.56	42.36	55.69	101.63	113.05	90.44
Reflected propagation	R4	13.80	41.89	58.31	111.62	152.32	133.52
	R3	10.95	27.61	36.89	68.07	88.54	96.63
	R2	9.28	19.04	26.66	45.93	58.07	67.35
	R1	7.85	14.76	15.95	20.23	24.99	32.84

**Table 3:** Peak-to-peak voltage measured (in mV) at R0 to R90 in the UGWT run following procedure P1 and procedure P2 with 10 PWAS and different delays in emission.

Process		R90	R75	R60	R45	R30	R15	R0
	P1	161.36	30.94	27.13	19.28	19.52	32.13	66.16
P2 with delay (μs)	0.00	121.14	107.10	23.09	16.66	22.13	24.99	73.30
	0.33	123.76	99.96	20.47	13.80	15.47	21.18	47.84
	0.50	126.14	88.77	19.28	13.57	16.66	17.61	41.89
	0.67	125.19	82.35	21.42	16.90	14.28	16.66	40.94
	1.00	129.95	93.30	31.18	20.71	26.42	20.23	50.93
	1.33	111.38	134.71	38.79	24.75	21.42	24.75	55.22
	1.60	117.81	134.95	68.31	30.70	27.85	40.22	80.68
	1.67	113.29	98.06	91.15	46.89	38.08	48.55	84.01
	1.80	116.86	70.92	122.81	87.35	64.74	75.92	95.91
	2.00	126.14	92.82	72.35	80.44	78.78	75.21	94.49
	2.33	136.85	131.14	31.18	32.37	34.27	42.84	114.95
